# Web Resources for Metagenomics Studies

**DOI:** 10.1016/j.gpb.2015.10.003

**Published:** 2015-11-18

**Authors:** Pravin Dudhagara, Sunil Bhavsar, Chintan Bhagat, Anjana Ghelani, Shreyas Bhatt, Rajesh Patel

**Affiliations:** 1 Department of Biosciences, Veer Narmad South Gujarat University, Surat 395007, India; 2 Department of Microbiology, Shree Ramkrishna Institute of Computer Education and Applied Sciences, Surat 395001, India; 3 Department of Life Sciences, Hemchandracharya North Gujarat University, Patan 384265, India

**Keywords:** Metagenomics, Metagenomes, Web resources, Software tools, Synthetic metagenome

## Abstract

The development of next-generation sequencing (NGS) platforms spawned an enormous volume of data. This explosion in data has unearthed new scalability challenges for existing bioinformatics tools. The analysis of metagenomic sequences using bioinformatics pipelines is complicated by the substantial complexity of these data. In this article, we review several commonly-used online tools for **metagenomics** data analysis with respect to their quality and detail of analysis using simulated **metagenomics** data. There are at least a dozen such **software tools** presently available in the public domain. Among them, MGRAST, IMG/M, and METAVIR are the most well-known tools according to the number of citations by peer-reviewed scientific media up to mid-2015. Here, we describe 12 online tools with respect to their web link, annotation pipelines, clustering methods, online user support, and availability of data storage. We have also done the rating for each tool to screen more potential and preferential tools and evaluated five best tools using **synthetic metagenome**. The article comprehensively deals with the contemporary problems and the prospects of **metagenomics** from a bioinformatics viewpoint.

## Introduction

Metagenomics has become an emerging and indispensable field of microbial ecology since the first use of the concept in 1998 [1]. It deals with the study of genetic material recovered directly from environmental samples. Originally, metagenomics studies depended on the cultivation of clonal cultures followed by functional expression screening [1], which are not able to represent the total community profile and may overlook the vast majority of the microbial biodiversity [2]. The advent of next-generation sequencing (NGS) technologies has made it possible to perform sequencing-based metagenomic analyses. NGS platforms allow researchers to retrieve high-throughput data at substantially lower cost, fueling the development of sequence-based metagenomics analyses aimed at decoding the entire microbial community [3].

Two distinct metagenomics approaches are commonly used: the first is referred to as marker-gene metagenomics or targeted metagenomics, and the second is shotgun metagenomics. The former involves the use of marker genes (including conserved markers such as 16S rRNA [4], [5], 18S rRNA, and ITS [6]) and an amplicon sequencing method to reassemble the taxonomic structure of a microbial community [7], [8]. For the latter approach, the random nature of shotgun sequencing ensures adequate coverage to assess the entire community’s structure [9], [10] and opens new avenues for discovering structural and functional novelties [11]. At present, thousands of marker-gene assisted and shotgun metagenomics projects have been undertaken, comprising millions of samples available in the public domain. After sequencing of a metagenomics sample, the primary task is to analyze the vast amount of data in order to determine the identities and roles of the microbial species present in the sample [12]. Several offline tools are available for classifying metagenomic reads against known reference datasets [13]. However, the assembly of metagenomic sequence data and the identification of the operational taxonomic units (OTUs) are major technical challenges that persist in metagenomic data analysis pipelines [14]. Moreover, the high complexity of metagenomics data is a critical barrier in analyzing these metadata using stand-alone tools. On the other hand, various web-based workflows may be able to offer in-depth exploration without the need for highly configured computers.

**Figure 1:**
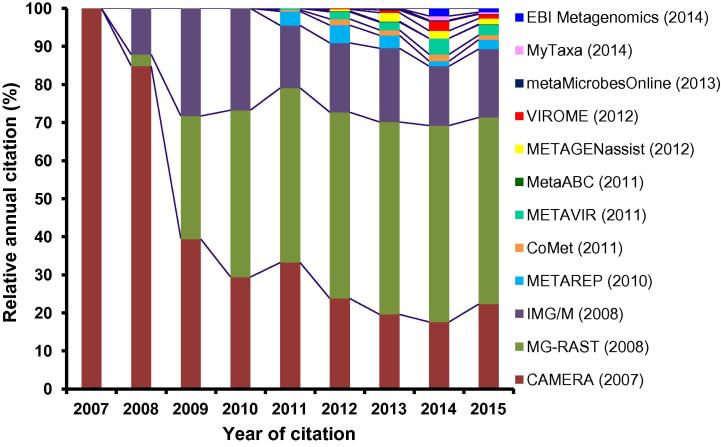
**Relative citation of the metagenomics software tools from articles published in peer-reviewed scientific journals by October 2015** Year in the bracket in the legend box indicates the year of original release of the respective tool. Citation of each tool was tracked from Google Scholar. Total citation of all tools is considered as 100% in each year and relative percentage of citations of each tool per year was calculated in relative to the total citations of all tools in the respective year.

In this article, we review the most useful online software tools for metagenomics data analysis, including a brief description of how they work in the course of the analysis. More importantly, we have rated the tools on the basis of overall users’ experience in five vital criteria: (1) ease of data uploading, (2) availability of online user support, (3) spectrum of data analyses available, (4) number of citations in scientific literature, and (5) size of the stored data (Figure 1, Table 1). While such an evaluation can never be completely thorough, we nevertheless hope that our evaluation of these web resources for metagenomics studies will provide vital information for budding scientists, assisting them to make the best choice for their preferred applications.

## MG-RAST

The software package Metagenomics Rapid Annotation using Subsystem Technology (MG-RAST) is a user-friendly open-source server initially constructed on the SEED project (http://www.theseed.org/) [15] framework for metagenomics studies [16], [17], [18]. Debuting in 2007, MG-RAST was one of the earliest online metagenomics analysis tools [16]. A new version (GitHub embedded version 3.6) is currently available, which does not entirely depend on SEED technology but uses the SEED subsystem instead as a preferred data source, to enable taxonomic and functional classification of metagenomes. The new version is encapsulated and separated from the data store, allowing far greater scalability and much faster computation than previous versions. Currently, MG-RAST contains 215,773 metagenomic datasets, which have been accessed by more than 12,000 registered users. Other than data access, registered users can also submit their own raw metagenomic data in FASTA, FASTQ, and SFF formats along with detailed sample information. The uploaded data are processed by a multi-step workflow that includes quality control, automated annotation, and analysis of prokaryotic metagenomic shotgun samples as well as targeted samples. Automated annotation is done using numerous reference datasets. The server supports a variety of user-driven analyses, including phylogenetic, functional, metabolic, and comparative analyses of two or more metagenomes. MG-RAST also provides the facility to select a database for quantitative insights into microbial populations based on sequencing data. It also offers the ability to output data in multiple clustering forms that are downloadable in flat text format or to export data as FASTA and QIIME files. Registered users have the right to publish their data openly or keep data private and can share data among multiple users with protected confidentiality. In these ways, MG-RAST offers great flexibility in analysis, privacy, and sharing of data. Therefore, it is the most reviewed and explored tool in use today [8], [13], [19]. So far, more than 2000 metagenome projects and over 13,000 metagenomic datasets have been uploaded to the MG-RAST servers each month of 2015, indicating the great popularity of the tool.

## IMG/M

The software referred to as the Integrated Microbial Genomes and Metagenomes (IMG/M) is a data storage, management, and analysis system for metagenomes hosted by the Joint Genome Institute (JGI) of U.S. Department of Energy (DOE) [20]. IMG/M integrates metagenome datasets with isolated microbial genomes from the IMG system. It is a domain-specific tool of IMG compatible with sequencing data from microbial communities. IMG/M allows for combined analyses with all available draft and complete genomes, plasmids, and viruses in the public domain [21], [22]. It provides free support for genomic and metagenomic data annotation, integration, and comparative analyses of integrated genomic and metagenomics data. The data content and analytical tools are updated continually. As of January 2015, IMG/M contained a total of 5234 metagenome datasets with billions of genes, out of which 3193 metagenome datasets were publicly available. More than 13,945 registered users from 95 countries had gained access as of October 2015. Registered users can access IMG/M data content and analytical tools. Registered users can submit data online and maintain data’s privacy for two years before data move to the public domain. Furthermore, analysis and downloading of metagenomics datasets via JGI’s portals is also possible for registered users. Pre-processing, quality control, and annotation of input data are performed by JGI’s metagenome annotation system. The stored IMG/M data are annotated using multiple reference datasets to conduct analyses in three tiers: (i) phylogenetic composition, (ii) functional or metabolic potential within individual microbiomes, and (iii) comparisons across microbiomes. IMG/M provides support for such analyses by integrating metagenome datasets with isolated microbial genomes from the IMG system [23]. IMG/M provides the output data in multiple-cluster forms, which facilitates its use as an online tool for environmental and organismal metagenomics analysis. In addition to providing integrated microbial metagenomes, IMG/M has developed the customized IMG/M-HMP (Human Microbiome Project; http://www.hmpdacc.org), which provides analysis tools for the annotation of HMP-generated metagenome datasets in the reference of all publicly-available genomes and metagenomics samples in IMG [24]. IMG/M attracts increasing number of researchers due to its in-depth descriptive analyses of metagenomes and genome extraction through binning of metagenomes.

## METAREP

Metagenomics Reports (METAREP) is openly available software for comparative metagenomics analysis. It is a well-known open browser for annotation, analysis, and comparison of short reads or assemblies of metagenomics data [25]. To upload input data, it requires only basic programming knowledge and the skill to operate the stand-alone version of METAREP. Users can simultaneously link a minimum of two datasets at various functional and taxonomic levels for comparative analyses through applying multiple clustering methods. For each of these clustering methods, METAREP provides a download option to export tab-delimited files for downstream analysis. The software integrates highly-scalable platforms Solr/Lucene, R, and CAKEPHP to compare huge volumes of metagenomic annotations. METAREP has been developed and maintained under utmost privacy by the J.C. Venter Institute (JCVI), and it is hosted at GitHub. Users are encouraged to suggest new features of interest for improving and updating the application. Recently, JCVI has updated the software with a better annotation process, clustering procedure, and input format. These improvements are practically demonstrated by the comparative analysis of shotgun metagenomics data from the HMP (http://www.hmpdacc.org/) [26].

## CoMet

CoMet is a freely accessible tool for comparative metagenomics analysis. It is the right pipeline for rapid analysis of metagenomic short-read data [27]. CoMet can easily handle metagenomics data generated by the 454 and Ion Torrent platforms. For the comparative analysis of metagenomic data, it accepts up to 20 metagenomic DNA sequencing files in multiple FASTA format as input; however zip files may also be submitted for faster uploading. Data sized up to 500 MB can be uploaded at a time for analysis through the easy-to-use submission interface. CoMet is specifically designed to compare the functional or metabolic profiles of multiple metagenomes in terms of Pfam protein domains and the associated Gene Ontology (GO) terms. CoMet combines ORF-finding and subsequent assignment of protein sequences to Pfam domain families with a comparative statistical analysis, providing a quick overview of putative functional differences in a set of metagenomic samples. Registration is not mandatory for job submission. The output comprises downloadable text files and also provides figures and a matrix of the statistical results. It has been the most used tool for comparative metagenomics study since 2011.

## METAGENassist

METAGENassist is a freely available webserver for comparative metagenomic analysis. It can provide a bacterial census from diverse biomes and performs comprehensive multivariate statistical analyses on the data [28]. The server accepts the CSV file format along with a wide variety of common formats generated by online and standalone metagenomic analysis tools such as mothur, MG-RAST, QIIME, and MEGAN. It is able to analyze marker-gene assisted as well as shotgun metagenomic data. METAGENassist is the only webserver to perform taxonomic-to-phenotypic mapping of the submitted data. Phenotypic information covering nearly 20 significant functional categories is automatically generated from the input data. The server uses univariate and multivariate data processing and follows a multi-step workflow to cluster data into different layouts. Output is available in annotated colorful tables and graphs, which can be downloaded in PNG or PDF formats.

## MetaABC

MetaABC is an integrated metagenomics platform for data adjustment, binning, and clustering. Its primary focus is data filtration and normalization techniques for improving taxonomic assignment in metagenomic analysis [29]. MetaABC was developed with compatibility for the metagenomic data generated by both NGS and Sanger platforms. Currently, more than 50 metagenomic datasets have been stored in MetaABC. Users can simultaneously upload 2–20 SFF, FASTA, or FASTQ files for analysis in My MetaABC. After the uploading of data files, the user can select and execute all the steps for abundance profiling of microbes. The final outputs are delivered to a user-friendly interface and can be retrieved for downloading in different visualization formats. Although MetaABC has advantages, such as integrating a hierarchical clustering program for comparative analysis of metagenomes, it has limitations as it is not applicable for functional and metabolic profiling of metagenomes. Moreover, its data privacy is limited because unregistered use can also access the data, and sharing feature is not available. Furthermore, it does not accept large (>2 MB) input files. Instead, MetaABC provides a stand-alone version to deal with larger datasets. It has been freely available in the public domain for the last four years, though its citations are low and it does not seem to be gaining enough attention in the scientific field.

## MyTaxa

MyTaxa is the latest novel platform for the taxonomic assignment of metagenomic sequences with high accuracy. It has homology-based bioinformatics framework to classify metagenomic and genomic data with extraordinary precision [30]. MyTaxa employs all genes present in an unidentified sequence as classifiers, weighting each gene on the basis of its pre-computed classifying power at a specified taxonomic level and frequency of horizontal gene transfer [30]. MyTaxa is specifically applicable to consigning and classifying unknown genomic and metagenomic sequences. It consists of a standalone indexed database that is freely accessible and downloadable from MyTaxa’s website. The database contains parameters for the gene clusters that are employed in the online analysis for taxonomic classification. The database is regularly updated to achieve high sensitivity and specificity in taxonomic analyses by incorporating additional reference gene sequences that are available through isolated genome or sequencing projects. The tool is scalable to analyze large dataset as well as compatible with any new, faster algorithms besides homology search algorithms. In the web server, users can start MyTaxa analyses by supplying two files, a standard GFF file and a tabular output file. MyTaxa is equipped with a unique classification system based on the genome-aggregate average amino acid identity (AAI) concept. This classification system substantiates MyTaxa as an appropriate tool for determining the degree of novelty of unknown metagenomic sequences. However, due to the absence of representative sequences for diverse species in the public domain, taxonomic assignment of the lowest taxa (*i.e.*, species) is a challenging task for MyTaxa. Moreover, its utility is hampered by the inability to resolve the functional or metabolic profiling of metagenomes. Finally, the BLASTx-translated metagenomic protein file is a prerequisite for operating the analytical tools, which turns out to be a key barrier for the new learners.

## metaMicrobesOnline

The metaMicrobesOnline software is a free web portal for the phylogenetic study of genes from microbial genomes and metagenomes. The metaMicrobesOnline analysis pipeline starts with contig assembly, gene calling, and translation into protein sequences, followed by scanning the sequence against recognized gene and protein domain families [31]. Outputs of the scanning are used to add genes from the metagenomics data to a multiple sequence alignment for each gene family, followed by construction of phylogenetic trees for each gene/domain family using FastTree-2. Users can select the optimal approach for their data, from assembly and gene calling to gene-tree analysis. Genome-context browsing is available, combining metagenomic contigs and isolated genomes. Therefore, users can determine the contiguously-sequenced genome and search functionally-associated genes. metaMicrobesOnline contains a secured database of 155 metagenomes including 123 ecological and 32 host-associated metagenomes. It has been integrated with the MicrobesOnline (http://www.microbesonline.org) isolated genomes since July 2010, which holds thousands of genome sequences from all three domains of life [32]. However, currently this tool is not being used widely and has not been frequently cited due to a lack of regular updates, the limited range of analyses, and restricted submission of new metagenomes.

## EBI Metagenomics

EBI Metagenomics was the first resource developed in Europe by EMBL-EBI in 2011 for taxonomic, functional, and comparative metagenomics analysis [33]. It contains QIIME-embedded tools for data management, analysis, storage, and sharing of metagenomes. Users must register to upload raw nucleotide reads that can remain private up to 2 years upon request, and reads are eventually stored with unique accession numbers in the European Nucleotide Archive (ENA). The uploaded datasets, along with essential details, comply with the Genomic Standards Consortium (GSC) and Minimum Information about any (X) Sequence (MIxS) for validation, so that the unambiguous data can be achieved and reused by the scientific community. Furthermore, users can submit raw sequencing reads generated from any NGS platforms through ENA’s Webin tool or ISAcreator. The EBI Metagenomics analysis pipeline includes trimming and quality checks to remove artifacts and streamline of the data. The processed reads are analyzed by rRNAselector and QIIME to predict taxa, followed by functional assignment by a composite protein database called InterProScan. EBI Metagenomics is well suited for the analysis of shotgun and marker-gene metagenomes. At present, it is mainly focused on shotgun metagenomes; however, the use of rRNASelector for the extraction of rRNA from shotgun metagenomic datasets is a key feature for analyzing marker-gene metagenomes. The results of analyses are easily accessible via the EBI Metagenomics web interface and are downloadable in a variety of forms compatible with further analyses using online or standalone tools. Rapid comparative taxonomic and functional profiling of metadata is also made possible using EBI Metagenomics. The comparative analysis allows extraction and identification of common and unique information among various metagenomes. Due to the multiple advantages offered by EBI Metagenomics, it has quickly become a favorite tool among beginners. EBI Metagenomics is constantly updating the analysis pipeline with the development of additional analysis and visualization tools. Currently, it endeavors to establish a common collaborative platform for NGS computational setups. In near future, multi-omics data analysis should also be possible through the EBI Metagenomics platform.

## CAMERA

The Community Cyberinfrastructure for Advanced Marine Microbial Ecology Research and Analysis (CAMERA) was developed in 2007 with the aim of monitoring microbial communities of the ocean and their response to environmental changes [34]. In 2010, CAMERA expanded its mission to include all metagenomics data derived from different biomes by dropping the word “marine” from its original name [35]. Currently, the Data Distribution Center (DDC) of CAMERA is no longer available at the original domain (http://camera.calit2.net), but has been relocated to the iMicrobe Project (http://data.imicrobe.us), which holds 128 projects and 2660 samples as of October 2015. Like MG-RAST and IMG/M, CAMERA is a prominent large-scale database that processes, shares, and secures metagenomic datasets. The sharing of data and computed results is currently under the auspices of the GSC. CAMERA permitted the dataset’s publication and was the first web tool to support the GSC’s Minimal Information checklists for metadata. To submit data, users are required to register in the iPlant cyberinfrastructure through the Discovery Environment (DE) web interface. Registered users normally are allotted 100 GB of virtual space, and an additional allocation of up to 1 TB can be granted upon request. The uploading and importing of huge datasets (>1.9 GB) within the DE is carried out either by Cyberduck for Mac and Windows users or by iDrop Desktop (iCommands) for LINUX users. Alternatively, small data files can be uploaded and imported within the DE using a simple URL. CAMERA’s fundamental task is to create a rich, distinctive data repository and bioinformatics tool resource that overcomes the unique challenges of metagenomics such as complexity, heterogeneity, and fragmented data. The database includes environmental metagenomic and genomic sequence data, associated environmental parameters, pre-computed search results, and software tools to support powerful cross-analysis of environmental samples. It holds a large number of samples from across the world; however, since 2011, its use and resulting citations have consistently decreased because of the technically lengthy and complicated data uploading process. The descriptive analysis of metagenomes is also not user-friendly. However: recently, it is incorporated into QIIME and offers rapid online cloud computing service with greater user involvement and tractability during the analysis process.

## METAVIR

METAVIR is a freely available webserver exclusively designed to annotate the raw and assembled viral metagenomic sequences [36]. Recently, a new framework, METAVIR 2, has been released that allows for comprehensive virome analysis [37]. The server is useful for exploring viral diversity from metagenomes and undertakes comparative analyses through pre-computed phylogenies of viral marker genes, rarefaction curves, and multivariate analyses constructed on *k*-mer signatures and BLAST-based comparisons. METAVIR 2 can handle assembled viromes made up of thousands of large contigs. As of October 2015, METAVIR holds 335 virome projects that comprise more than 64 million sequences from diverse samples. Registered users can upload their sequence datasets only in FASTA file in a private space. However, users can also submit large data with compressed files in zip, gzip, or tar.gz format. The output can be visualized in different interactive and dynamic forms, *i.e.*, tables, phylogenetic trees, recruitment plots, and maps. METAVIR has an easy-to-use submission interface and offers rapid in-depth virome comparison along with data privacy, which are key features that make it the most cited virome analysis pipeline.

## VIROME

Viral Informatics Resource for Metagenome Exploration (VIROME) is a web-based application specifically designed for the analysis of metagenomic data collected from viral assemblages occurring within different environmental contexts. It emphasizes the classification of metagenome viral sequences and putative ORFs based on homology search results against both known and environmental sequences [38]. Submission of sequence data to VIROME is initiated through the web-application interface. After filling out a form, the user is contacted by Email correspondence with further instructions regarding sequence data transfer. It accepts multiple file formats, including FASTA, QUAL, FASTQ, and SFF. Metagenome libraries submitted for analysis at VIROME are blasted against the MetagenomesOnline database (MgOl), with a graphical interface and links to various protein metadata enabling exploration of the results. The VIROME workflow consists of two consecutive steps, sequence quality screening and sequence analysis against the UniVec database, followed by three parallel steps that include ORF-calling using MetaGeneAnnotator, known protein identification, and environmental protein characterization by subject databases such as MGOL and UniRef 100. Sequence quality trimming is run using a workflow management system called Ergatis. Data from the sequence processing and BLAST analysis components are stored in a back-end MySQL database. An Adobe ColdFusion server handles communication between the MySQL database and the VIROME web application. VIROME offers downloadable output that can be used as input data for multivariate analyses using third-party tools. The main hurdle to its widespread use is the length of time needed to analyze even a small-sized metagenome, usually several weeks to months.

## Analysis of synthetic metagenome

Synthetic metagenome was analyzed using five selected tools to demonstrate the efficiency and analysis time course. Synthetic metagenome (Run accession: ERR340319) was downloaded from EMBL-EBI. It contains 32,782,167 bp incorporated in 90,385 raw sequences generated from 454 GS FLX Titanium sequencing. This synthetic metagenome was uploaded into MG-RAST, IMG/M, EBI Metagenomics, CoMet and METAVIR that were selected based on rating criteria for taxonomic assessment using online support provided by the software developers. The result showed the numbers of different microbial families reported from each tool are not exactly similar due to the different annotation pipelines used by these tools. Moreover, the analysis time required by the each tool was also varied, because analysis depends on the number of running job in server and length of input sequences (Table 2). Though the remaining tools provided online help for analysis, it was not feasible to complete the data submission and taxonomic analysis of such a large synthetic metagenome within a short period of time by beginning users.

## Concluding remarks

Structural and functional microbial profiling has rapidly become commonplace, thanks to the advent of NGS platforms. Additionally, the comparative metagenomics of various biomes quickly enlarged the field of microbial ecology. To decode the ecosystem comprehensively, new tools, frameworks, and hypotheses will be required for analysis, storage, visualization, and sharing of the dataset. A single platform is therefore not adequate for conducting holistic metagenomics analyses. Longer-read sequences, accurate assembly, and annotation pipelines are anticipated developments from impending metagenomics study. The present review has given an overview of the prevailing metagenomics analysis tools, and we expect more advanced software tools from their original developers in the near future. This review may serve as an atlas for rising researchers to choose the most appropriate web-based platforms for metagenomic data analysis. It is specifically intended to aid those organizations that have limited computational resources with which to analyze their data.

## Competing interests

The authors have declared no competing interests.

## Figures and Tables

**Table 1:** Main online software tools for metagenomics studies

**Name**	**Weblink**	**Inbuilt annotation pipelines**	**Clustering methods**	**Online user support**	**Data storage**	**Rating**	**Refs.**
MG-RAST	https://metagenomics.anl.gov/	SEED subsystem, COG, KO, NOG, eggNOG, M5RNA, KEGG, TrEMBL, SEED, PATRIC, SwissProt, GenBank, RefSeq	HM, PT, BC, T	Blog and manual	215,773 metagenome dataset and 30,589 public metagenomes		[8], [11]

IMG/M	http://img.jgi.doe.gov/m	COG, KOG, KEGG, KO, Pfam, TIGRfam, TIGR, MetaCyc, GO	T, PC, PT, RP, HM	User guide, user forum, standard operating procedure	32,802 genome and 5234 metagenome dataset		[14], [15], [16], [17], [18]

METAREP	http://jcvi.org/metarep/	GO, NCBI Taxonomy	T, HM, HCP	User manual, demo video	No storage		[19], [20]

CoMet	http://comet.gobics.de/	Pfam, GO	T, DG, BC, DM	Online help	No storage		[21]

METAGENassist	http://www.metagenassist.ca/METAGENassist/	BacMap, GOLD, NCBI Taxonomy, PubMed	DG, HM, KM, SOM	Tutorials, FAQs, data examples	No storage		[22]

MetaABC	http://metaabc.iis.sinica.edu.tw/	Database of reference genomes (NCBI)	HM, BC, PC	Online help	52 dataset		[23]

MyTaxa	http://enve-omics.ce.gatech.edu/mytaxa/	Database of reference genes and genomes (NCBI)	PT, BC	FAQs, examples	No storage		[24]

metaMicrobesOnline	http://meta.microbesonline.org/	TIGRfam, COG, Pfam	T, PT	Guide, tutorial, help through email	155 metagenome and 3527 genome dataset		[25], [26]

EBI Metagenomics	https://www.ebi.ac.uk/metagenomics/	RDP, Greengenes database, InterPro protein signature database	T, PC, BC, HM, SC, PCA	Training, online support by email and Twitter	141 projects and 5800 dataset		[27]

CAMERA	http://camera.calit2.net/	FragGeneScan, MetaGene, COG, Pfam, TIGRfam, GO, KEGG	T, PC, DG	Tutorials, video tutorials, online manual	128 projects and 2660 samples		[28], [29]

METAVIR	http://metavir-meb.univ-bpclermont.fr/	Pfam, RefSeq virus database	HM, DG, RP, PC	Video tutorial, guide, FAQs, online contact	170 viral metagenomic dataset and 335 projects		[30], [31]

VIROME	http://virome.dbi.udel.edu/	SEED, ACLAME, COG, GO, KEGG, MGOL, UniRef 100	T, PC, TDD	Tutorial videos	466 libraries containing 24,386,816 reads		[32]

*Note:* Darkness of the grayscale bars in the rating column indicates higher importance and usefulness. Rating is based on five different criteria represented with a gray scale from left to right: (i) easiness in data uploading, (ii) availability of online user support, (iii) spectrum of analysis, (iv) citation, and (v) stored data size. HM, heatmap; PT, phylogenetic tree; BC, bar chart; T, tabulation; PC, pie chart; RP, recruitment plot; HCP, hierarchical cluster plot; DM, distance matrix; DG, dendrogram; KM, K-means; SOM, self-organizing map; TDD, tab-delimited data; SC, stacked column; PCA, principal component analysis. Data storage was obtained from the respective website in October 2015.

**Table 2:** Result of synthetic metagenome analysis using the five selected tools

**Tool**	**Total No. of microbial families identified**	**Analysis time course (h)**
MG-RAST	98	05
IMG/M	95	96
EBI Metagenomics	81	04
CoMet	172	05
METAVIR	100	72

*Note:* Tools were selected based on rating criteria for taxonomic assessment using online support provided by the software developers.

## References

[1] Handelsman J., Rondon M.R., Brady S.F., Clardy J., Goodman R.M. (1998). Molecular biological access to the chemistry of unknown soil microbes: a new frontier for natural products. Chem Biol.

[2] Daniel R. (2005). The metagenomics of soil. Nat Rev Microbiol.

[3] Thomas T., Gilbert J., Meyer F. (2012). Metagenomics – a guide from sampling to data analysis. Microb Inform Exp.

[4] Dudhagara P., Ghelani A., Patel R., Chaudhari R., Bhatt S. (2015). Bacterial tag encoded FLX titanium amplicon pyrosequencing (bTEFAP) based assessment of prokaryotic diversity in metagenome of Lonar soda lake, India. Genom Data.

[5] Ghelani A., Patel R., Mangrola A., Dudhagara P. (2015). Cultivation-independent comprehensive survey of bacterial diversity in Tulsi Shyam Hot Springs, India. Genom Data.

[6] Dudhagara P., Ghelani A., Bhavsar S., Bhatt S. (2015). Metagenomic data of fungal internal transcribed Spacer and 18S rRNA gene sequences from Lonar lake sediment, India. Data Brief.

[7] Carlos N., Tang Y.W., Pei Z. (2012). Pearls and pitfalls of genomics-based microbiome analysis. Emerg Microbes Infect.

[8] Oulas A., Pavloudi C., Polymenakou P., Pavlopoulos G.A., Papanikolaou N., Kotoulas G. (2015). Metagenomics: tools and insights for analyzing next-generation sequencing data derived from biodiversity studies. Bioinform Biol Insights.

[9] Patel R., Mevada V., Prajapati D., Dudhagara P., Koringa P., Joshi C.G. (2015). Metagenomic sequence of saline desert microbiota from wild ass sanctuary, Little Rann of Kutch, Gujarat, India. Genom Data.

[10] Mangrola A.V., Dudhagara P., Koringa P., Joshi C.G., Patel R.K. (2015). Shotgun metagenomic sequencing based microbial diversity assessment of Lasundra hot spring, India. Genom Data.

[11] Singh A.H., Doerks T., Letunic I., Raes J., Bork P. (2009). Discovering functional novelty in metagenomes: examples from light-mediated processes. J Bacteriol.

[12] Ounit R., Wanamaker S., Close T.J., Lonardi S. (2015). CLARK: fast and accurate classification of metagenomic and genomic sequences using discriminative k-mers. BMC Genomics.

[13] Mandal R.S., Saha S., Das S. (2015). Metagenomic surveys of gut microbiota. Genomics Proteomics Bioinformatics.

[14] Behnam E., Smith A.D. (2014). The Amordad database engine for metagenomics. Bioinformatics.

[15] Overbeek R., Begley T., Butler R.M., Choudhuri J.V., Chuang H.Y., Cohoon M. (2005). The subsystems approach to genome annotation and its use in the project to annotate 1000 genomes. Nucleic Acids Res.

[16] Meyer F., Paarmann D., D’Souza M., Olson R., Glass E.M., Kubal M. (2008). The metagenomics RAST server – a public resource for the automatic phylogenetic and functional analysis of metagenomes. BMC Bioinformatics.

[17] Overbeek R., Olson R., Pusch G.D., Olsen G.J., Davis J.J., Disz T. (2014). The SEED and the Rapid Annotation of microbial genomes using Subsystems Technology (RAST). Nucleic Acids Res.

[18] Wilke A., Bischof J., Harrison T., Brettin T., D’Souza M., Gerlach W. (2015). A RESTful API for accessing microbial community data for MG-RAST. PLoS Comput Biol.

[19] Sun Q., Liu L., Wu L., Li W., Liu Q., Zhang J. (2015). Web resources for microbial data. Genomics Proteomics Bioinformatics.

[20] Markowitz V.M., Ivanova N.N., Szeto E., Palaniappan K., Chu K., Dalevi D. (2008). IMG/M: a data management and analysis system for metagenomes. Nucleic Acids Res.

[21] Markowitz V.M., Chen I.M., Chu K., Szeto E., Palaniappan K., Grechkin Y. (2012). IMG/M: the integrated metagenome data management and comparative analysis system. Nucleic Acids Res.

[22] Markowitz V.M., Chen I.M., Chu K., Szeto E., Palaniappan K., Pillay M. (2014). IMG/M 4 version of the integrated metagenome comparative analysis system. Nucleic Acids Res.

[23] Markowitz V.M., Chen I.M., Palaniappan K., Chu K., Szeto E., Grechkin Y. (2012). IMG: the Integrated Microbial Genomes database and comparative analysis system. Nucleic Acids Res.

[24] Markowitz V.M., Chen I.M., Chu K., Szeto E., Palaniappan K., Jacob B. (2012). IMG/M-HMP: a metagenome comparative analysis system for the human microbiome project. PLoS One.

[25] Goll J., Rusch D.B., Tanenbaum D.M., Thiagarajan M., Li K., Methe B.A. (2010). METAREP: JCVI metagenomics reports – an open source tool for high-performance comparative metagenomics. Bioinformatics.

[26] Goll J., Thiagarajan M., Abubucker S., Huttenhower C., Yooseph S., Methé B.A. (2012). A case study for large-scale human microbiome analysis using JCVI’s metagenomics reports (METAREP). PLoS One.

[27] Lingner T., Asshauer K.P., Schreiber F., Meinicke P. (2011). CoMet – a web server for comparative functional profiling of metagenomes. Nucleic Acids Res.

[28] Arndt D., Xia J., Liu Y., Zhou Y., Guo A.C., Cruz J.A. (2012). METAGENassist: a comprehensive web server for comparative metagenomics. Nucleic Acids Res.

[29] Su C.H., Hsu M.T., Wang T.Y., Chiang S., Cheng J.H., Weng F.C. (2011). MetaABC – an integrated metagenomics platform for data adjustment, binning and clustering. Bioinformatics.

[30] Luo C., Rodriguez-R L.M., Konstantinidis K.T. (2014). MyTaxa: an advanced taxonomic classifier for genomic and metagenomic sequences. Nucleic Acids Res.

[31] Chivian D., Dehal P.S., Keller K., Arkin A.P. (2013). MetaMicrobesOnline: phylogenomic analysis of microbial communities. Nucleic Acids Res.

[32] Dehal P.S., Joachimiak M.P., Price M.N., Bates J.T., Baumohl J.K., Chivian D. (2010). MicrobesOnline: an integrated portal for comparative and functional genomics. Nucleic Acids Res.

[33] Hunter S., Corbett M., Denise H., Fraser M., Gonzalez-Beltran A., Hunter C. (2014). EBI metagenomics—a new resource for the analysis and archiving of metagenomic data. Nucleic Acids Res.

[34] Seshadri R., Kravitz S.A., Smarr L., Gilna P., Frazier M. (2007). CAMERA: a community resource for metagenomics. PLoS Biol.

[35] Sun S., Chen J., Li W., Altintas I., Lin A., Peltier S. (2011). Community cyberinfrastructure for Advanced Microbial Ecology Research and Analysis: the CAMERA resource. Nucleic Acids Res.

[36] Roux S., Faubladier M., Mahul A., Paulhe N., Bernard A., Debroas D. (2011). METAVIR: a web server dedicated to virome analysis. Bioinformatics.

[37] Roux S., Tournayre J., Mahul A., Debroas D., Enault F. (2014). METAVIR 2: new tools for viral metagenome comparison and assembled virome analysis. BMC Bioinformatics.

[38] Wommack K.E., Bhavsar J., Polson S.W., Chen J., Dumas M., Srinivasiah S. (2012). VIROME: a standard operating procedure for analysis of viral metagenome sequences. Stand Genomic Sci.

